# Rapid Phenotypic and Metabolomic Domestication of Wild *Penicillium* Molds on Cheese

**DOI:** 10.1128/mBio.02445-19

**Published:** 2019-10-15

**Authors:** Ina Bodinaku, Jason Shaffer, Allison B. Connors, Jacob L. Steenwyk, Megan N. Biango-Daniels, Erik K. Kastman, Antonis Rokas, Albert Robbat, Benjamin E. Wolfe

**Affiliations:** a Tufts University, Department of Biology, Medford, Massachusetts, USA; b Tufts University, Department of Chemistry, Medford, Massachusetts, USA; c Vanderbilt University, Department of Biological Sciences, Nashville, Tennessee, USA; d Tufts University Sensory and Science Center, Medford, Massachusetts, USA; University of California, Berkeley

**Keywords:** *Penicillium*, cheese, evolution, mycotoxins, secondary metabolism, transcriptome, volatile organic compound

## Abstract

Fermented foods provide novel ecological opportunities for natural populations of microbes to evolve through successive recolonization of resource-rich substrates. Comparative genomic data have reconstructed the evolutionary histories of microbes adapted to food environments, but experimental studies directly demonstrating the process of domestication are lacking for most fermented food microbes. Here, we show that during adaptation to cheese, phenotypic and metabolomic traits of wild *Penicillium* molds rapidly change to produce domesticated phenotypes with properties similar to those of the industrial cultures used to make Camembert and other bloomy rind cheeses. Over a period of just a few weeks, populations of wild *Penicillium* strains serially passaged on cheese had reduced pigment, spore, and mycotoxin production. Domesticated strains also had a striking change in volatile metabolite production, shifting from production of earthy or musty volatile compounds (e.g., geosmin) to fatty and cheesy volatiles (e.g., 2-nonanone, 2-undecanone). RNA sequencing demonstrated a significant decrease in expression of 356 genes in domesticated strains, with an enrichment of many secondary metabolite production pathways in these downregulated genes. By manipulating the presence of neighboring microbial species and overall resource availability, we demonstrate that the limited competition and high nutrient availability of the cheese environment promote rapid trait evolution of *Penicillium* molds.

## Abstract (precis)

Industrial cultures of filamentous fungi are used to add unique aesthetics and flavors to cheeses and other microbial foods. How these microbes adapted to live in food environments is generally unknown as most microbial domestication is unintentional. Our work demonstrates that wild molds closely related to the starter culture Penicillium camemberti can readily lose traits and quickly shift toward producing desirable aroma compounds. In addition to experimentally demonstrating a putative domestication pathway for P. camemberti, our work suggests that wild *Penicillium* isolates could be rapidly domesticated to produce new flavors and aesthetics in fermented foods.

## INTRODUCTION

Fermented foods such as cheese, miso, sourdough, and sauerkraut are hybrid microbiomes where wild microbial species from the environment mix with domesticated microbes that are added as starter cultures. Microbes from natural ecosystems have the potential to adapt to these resource-rich environments, where they may rapidly evolve new traits and/or lose traits that are not maintained by selection (1). Previous evidence for microbial domestication in fermented foods comes from comparative genomic studies of food isolates and closely related wild strains (1–4). For example, in the fungus Aspergillus oryzae, which is used in the production of soy sauce, miso, and sake, both structural and regulatory genomic changes are correlated with the evolution of nontoxic and flavorful A. oryzae strains from a highly toxic ancestor (Aspergillus flavus) (2). The evolutionary origins of most domesticated microbes remain enigmatic in large part because domestication of microbes is usually unintentional and the processes driving microbial domestication have not been experimentally recreated (1).

**FIG 1:**
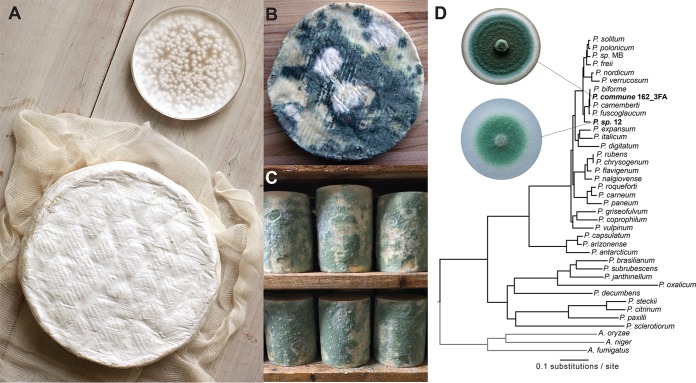
*Penicillium* molds in the cheese environment. (A) The white mold known as *Penicillium camemberti* (show in pure culture in the Petri dish) is used to make Camembert (shown), Brie, and other bloomy rind cheeses. (Photo by Adam DeTour and used with permission.) (B) Wild *Penicillium* molds (also known as non-starter molds) can contaminate cheeses during production. (C) Some natural rind cheeses are intentionally colonized by wild *Penicillium* molds. Shown here is *Penicillium* sp. strain 12, a strain used in the experiments in this paper, colonizing wheels of a blue cheese in a cave in the United States. (D) A phylogenomic tree of *Penicillium*. Strains used in this work are highlighted in bold. *Penicillium* sp. MB was also isolated from a natural rind cheese and sequenced as part of this work but was not used in the experiments described.

*Penicillium* species colonize the surfaces of aged cheeses around the world either as starter cultures that are intentionally added during the cheese making process (5, 6) or as non-starter *Penicillium* species that enter cheese production facilities from natural fungal populations (7–9). The white surface of Camembert and Brie is created by a variety of strains of the starter culture P. camemberti. This domesticated fungus is white, makes fewer conidia (asexual spores) than most wild *Penicillium* species, does not make detectable levels of mycotoxins, and produces desirable mushroom-like and fatty volatiles during cheese ripening (10–12) (Fig. 1A). In contrast, the putative ancestor P. commune and other closely related *Penicillium* species are generally greenish-blue, make large numbers of conidia, and produce mycotoxins and other undesirable volatiles that negatively impact cheese quality (Fig. 1B and C). Historical accounts suggest that white domesticated strains of *Penicillium* species were either directly isolated from French cheeses or produced in laboratories in France (13), but the potential domestication processes that generated these iconic cheese mold species have not been identified.

Here, we used experimental evolution to determine how wild *Penicillium* molds may be unintentionally domesticated in the cheese aging environment. We specifically determined how quickly *Penicillium* could evolve new phenotypes on cheese, how *Penicillium* traits change during domestication on cheese, and what properties of the cheese environment promote domestication of *Penicillium*. Using our cheese rind model (14, 15), we serially passaged populations of wild *Penicillium* and tracked phenotypic traits. We found that domesticated strains with substantially reduced mycotoxin levels, reproductive output, and pigment production rapidly emerged in these experimental populations. Volatile profiling and RNA sequencing (RNA-seq) demonstrated substantial remodeling of metabolism in domesticated strains. These findings illustrate the potential for rapid domestication of *Penicillium* and possibly other filamentous fungi in cheese caves around the world.

## RESULTS AND DISCUSSION

### Non-starter *Penicillium* species rapidly evolve novel phenotypes on cheese.

To experimentally evolve *Penicillium* on cheese, two non-starter *Penicillium* strains (Penicillium commune strain 162_3FA and *Penicillium* sp. strain 12) isolated from a cheese cave in Vermont in the United States were serially passaged on cheese curd agar (CCA) in the laboratory. These molds were isolated from a cheese aging facility that was colonized by the molds within the past 5 years. These cheese cave isolates have wild-type phenotypes (pigmented, high spore production, musty odors, and mycotoxin production) and are closely related to P. camemberti strains used in cheese production Fig. 1D; see also Fig. S1 in the supplemental material. At each passage, replicate populations were sampled to determine population size and frequency of domesticated phenotypes. We use the terms “domesticated phenotypes” and “evolved strains” instead of “mutants” throughout this work because we are currently unsure of the exact mechanism driving phenotypic evolution in this system. The term “mutant” implies that a specific genetic mechanism is causing phenotypic change, and that is currently unknown in this system. Colonies were considered domesticated phenotypes if they had altered surface color or texture indicating changes in pigment or spore production. To determine how competition from neighboring cheese microbes impacts the rate of phenotypic diversification, we serially passaged replicate populations alone (“*Penicillium* alone”) or in the presence of a mix of three competitors (“*Penicillium* + community,” including the yeast Debaryomyces hansenii and the bacteria Brachybacterium alimentarium and Staphylococcus xylosus) that commonly cooccur with *Penicillium* species in cheese rinds (14, 16, 17).

10.1128/mBio.02445-19.1 FIG S1 The genome-scale phylogeny of the genus *Penicillium.* (A) Concatenation phylogeny with section denominations. The two strains used in the experiments described in the text (indicated in bold) are placed within section *Fasciculata*. Furthermore, Penicillium commune strain 162_3FA is closely related to Penicillium biforme and Penicillium camemberti. The inset depicts phylogeny, with branch lengths representing substitutions per site. *Penicillium* sp. MB was isolated from a natural rind cheese at the same time as the other two strains and was sequenced as part of this work, but it was not used in the experiments described. (B) Comparison of concatenation-based (left) and coalescence-based (right) phylogenies reveals only one instance of incongruence. Specifically, whereas P. biforme is placed sister to P. commune 162_3FA in the concatenation analysis, coalescence supports P. camemberti as sister to P. commune 162_3FA. All internal branches received full support except the coalescence-inferred internal branch where *Exilicaulis* and *Lanata-divaricata* split, which received a local posterior probability value of 0.97. Branch lengths reflect substitutions/site for concatenation and coalescence units for the coalescence inferred phylogeny. Download FIG S1, DOCX file, 0.5 MB. Copyright © 2019 Bodinaku et al. 2019 Bodinaku et al. This content is distributed under the terms of the Creative Commons Attribution 4.0 International license.

**FIG 2:**
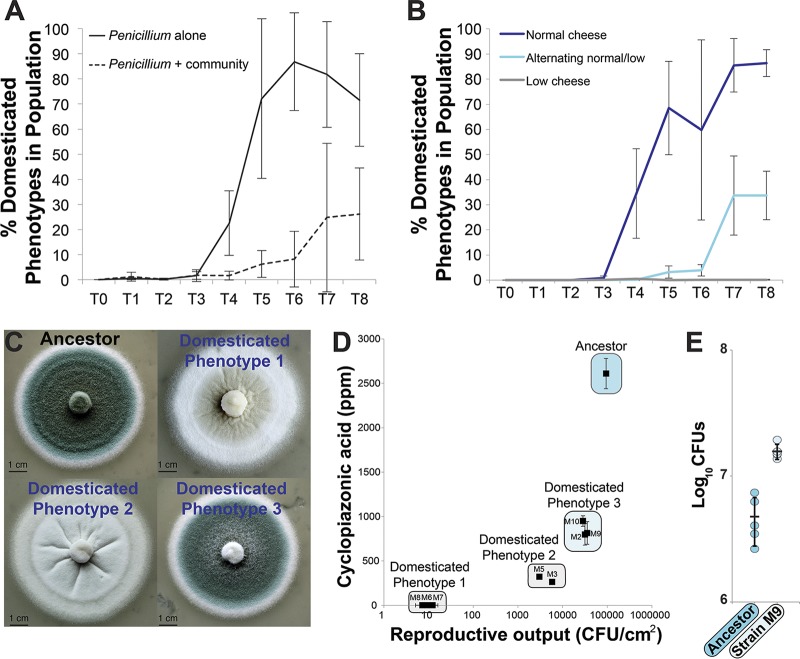
Experimental evolution of *Penicillium* on cheese. (A) Evolution of Penicillium commune strain 162_3FA on cheese curd alone (“*Penicillium* alone”) and in the presence of three competing cheese rind microbes (“*Penicillium* + community”; Staphylococcus xylosus, Brachybacterium alimentarium, and Debaryomyces hansenii). Lines connect points representing mean domesticated phenotype frequencies of four replicate populations, and error bars represent 1 standard deviation of the mean. “*Penicillium* + community” had a significantly lower domesticated phenotype frequency (repeated-measures ANOVA; see text for statistics). (B) Experimental evolution of *P. commune* strain 162_3FA in different cheese nutrient environments. “Normal cheese” = 10% cheese curd in agar medium. “Low cheese” = 1% cheese curd in agar medium. “Alternating normal/low” = alternating 10% and 1% cheese curd at each transfer. Both “Low cheese” and “Alternating normal/low” had significantly lower domesticated phenotype frequencies (repeated-measures ANOVA with Tukey’s HSD *post hoc* tests; see text for statistics). Lines connect points representing mean domesticated phenotype frequencies of four replicate populations, and error bars represent 1 standard deviation of the mean. The “low cheese” line is difficult to see because it is at 0%. (C) Morphology of four representative colony types. The ancestor phenotype was dark blue-green and dusty; domesticated phenotype 1 was white and flat; domesticated phenotype 2 was white and fuzzy/dusty; domesticated phenotype 3 was blue-green but had less intense coloration than the ancestor and a less fuzzy appearance. (D) Reproductive output and cyclopiazonic acid production of a range of strains isolated across the experimental evolution populations. Points are mean values, and error bars represent 1 standard deviation of the mean. Strains M5, M6, M7, and M8 had reduced reproductive compared to the ancestor (Dunnett’s test, *P < *0.05). All domesticated phenotypes had significantly reduced CPA production compared to the ancestor (Dunnett’s test, *P < *0.05). (E) Competition between ancestral *P. commune* strain 162_3FA and domesticated strain M9. Strain M9 outcompeted the ancestor after 10 days of growth on cheese curd agar. Points represent individual replicate competition communities, and the horizontal line indicates mean values. Error bars represent 1 standard deviation.

Within 4 weeks of serial passage on cheese, domesticated phenotypes began to emerge in our experimental *Penicillium* populations, reaching 71.5% of the population in the *Penicillium*-alone treatments by the end of the experiment (Fig. 2A; see also Table S1A and B). The presence of neighbors strongly inhibited domesticated phenotype frequencies in the *Penicillium*-plus-community treatments (mean of 26.2%, repeated-measures analysis of variance [ANOVA] *F*_1,6_ = 86.5, *P < *0.001) (Fig. 2A). The presence of the neighbors also resulted in decreased total population sizes, with an average of a 42% decrease in total CFU across the experiment (repeated-measures ANOVA *F*_1,6_ = 10.3, *P = *0.02) (Fig. S2). Similar patterns of phenotypic diversification alone and inhibition with neighbors were observed with *Penicillium* sp. strain 12 (Fig. S3A and B; see also Table S1C to D). These results suggest that cheese can promote the rapid phenotypic diversification of *Penicillium* molds and that biotic interactions in cheese rinds can inhibit this diversification.

10.1128/mBio.02445-19.2 FIG S2 Population size of Penicillium commune 162_3FA evolved alone and with a community of cheese microbes. Lines connect points representing mean CFUs from four replicate populations, and error bars represent 1 standard deviation of the mean. Total CFUs in the *Penicillium*-plus-community treatment were significantly different from those measured for the total measured for *Penicillium* alone (repeated-measures ANOVA *F*_1,6_ = 10.3, *P = *0.02). Download FIG S2, DOCX file, 0.1 MB. Copyright © 2019 Bodinaku et al. 2019 Bodinaku et al. This content is distributed under the terms of the Creative Commons Attribution 4.0 International license.

10.1128/mBio.02445-19.3 FIG S3 Experimental evolution of *Penicillium* sp. 12 alone and with a community of cheese rind microbes. (A) Population size of *Penicillium* sp. 12 evolved alone and with a community of cheese microbes. Lines connecting points represent mean domesticated phenotype frequencies of four replicate populations, and error bars represent 1 standard deviation of the mean. Total CFUs in the *Penicillium*-plus-community treatment were significantly different from those measured for *Penicillium* alone (repeated-measures ANOVA *F*_1,6_ = 16.8, *P* = 0.006). Lines connect points representing mean CFUs from four replicate populations, and error bars represent 1 standard deviation of the mean. (B) Domesticated phenotype frequency of *Penicillium* sp. 12 evolved alone and with a community of cheese microbes. Lines connect points representing mean domesticated phenotype frequencies of four replicate populations, and error bars represent 1 standard deviation of the mean. Domesticated phenotype frequencies in the *Penicillium*-plus-community treatment were significantly different from those measured for *Penicillium* alone (repeated-measures ANOVA *F*_1,6_ = 20.1, *P* < 0.005). Download FIG S3, DOCX file, 0.2 MB. Copyright © 2019 Bodinaku et al. 2019 Bodinaku et al. This content is distributed under the terms of the Creative Commons Attribution 4.0 International license.

10.1128/mBio.02445-19.7 TABLE S1 (A) Description of domesticated phenotype classes and specific isolates identified in the evolution of Penicillium commune strain 162_3FA. (B) Distribution of domesticated phenotypes across replicate populations and transfers in the experimental evolution of Penicillium commune 162_3FA. Data at the bottom show the number of colonies of ancestor or domesticated phenotypes counted from 10^−4^ dilution plates of experimental populations. T1, T2, etc. = transfer numbers. (C) Description of specific domesticated phenotype isolates identified in the evolution of *Penicillium* sp. strain 12. (D) Distribution of domesticated phenotypes across replicate populations and transfers in the experimental evolution of *Penicillium* sp. 12. Data at the bottom show the number of colonies of ancestor or domesticated phenotypes counted from 10^−4^ dilution plates of experimental populations. T1, T2, etc. = transfer numbers. Download Table S1, XLSX file, 0.1 MB. Copyright © 2019 Bodinaku et al. 2019 Bodinaku et al. This content is distributed under the terms of the Creative Commons Attribution 4.0 International license.

Cheese is rich in nutrients compared to the environments where *Penicillium* molds naturally occur (soils, leaves, etc.), with an abundance of carbon, nitrogen, and other resources stored in the protein casein (18). To determine if the high resource availability of cheese promotes the rapid domestication of *Penicillium* molds, we repeated the short-term experimental evolution experiment described above with the standard cheese curd (“normal cheese”), with a “low-cheese” treatment with 1/10 the normal amount of cheese curd, and with an “alternating cheese” treatment with alternating normal and 1/10 cheese curd at every other passage. The low-cheese treatment was designed to reduce total nutrient availability while maintaining pH and the levels of other environmental variables. The alternating treatment was designed to simulate alternating colonizations of a high-resource environment (cheese) and a low-resource environment (soil, wood) such as could occur in a cheese aging facility.

As with our first set of experiments, the normal cheese treatment resulted in the rapid evolution of domesticated phenotypes by the fourth week of the experiment, with a mean domesticated phenotype frequency of 89.0% (±12.2) across four replication populations at the end of the experiment (Fig. 2B). Both the alternating-cheese treatment and low-cheese treatment had significantly lower domesticated phenotype frequencies across the duration of the experiment (mean frequencies at end of experiment: alternating cheese = 73.0% ± 14.6%; low cheese = 0.16% ± 0.19% [repeated-measures ANOVA *F*_2,9_ = 149.6, *P < *0.0001]). As with the competition treatment described above, population sizes were significantly lower in the low-cheese treatment (repeated-measures ANOVA *F*_2,9_ = 105.1, *P* < 0.0001), which may explain the substantially suppressed rate of diversification (Fig. S4). These results suggest that the high-resource environment of cheese promotes the rapid trait evolution of *Penicillium*.

10.1128/mBio.02445-19.4 FIG S4 Population size of Penicillium commune 162_3FA evolved in different cheese nutrient environments. “Normal cheese” = 10% cheese curd in agar medium. “Low cheese” = 1% cheese curd in agar medium. “Alternating normal/low” = alternating 10% and 1% cheese curd at each transfer. The “Low cheese” treatment suppressed population size (repeated-measures ANOVA *F*_2,9_= 105.1, *P* < 0.0001, with Tukey’s HSD *post hoc* tests). Lines connect points representing mean CFUs from four replicate populations, and error bars represent 1 standard deviation of the mean. Download FIG S4, DOCX file, 0.2 MB. Copyright © 2019 Bodinaku et al. 2019 Bodinaku et al. This content is distributed under the terms of the Creative Commons Attribution 4.0 International license.

### Domestication of *Penicillium* on cheese leads to stable reductions in reproductive output, mycotoxin production, and pigmentation.

In our work described above, all strains that emerged with altered colony morphotypes were grouped together to obtain overall rates of phenotypic evolution in different biotic and abiotic environments. To provide a finer-scale analysis of how reproductive and metabolic traits shifted during adaptation to cheese, we measured the reproductive output and mycotoxin production of representative strains of P. commune 162_3FA that spanned the spectrum of domesticated colony phenotypes (Table S1A and B). Reproductive output was measured as the number of CFU produced per unit area of a fungal colony and included both spore and hyphal propagules. We also measured production of the mycotoxin cyclopiazonic acid (CPA) by ancestral and evolved strains grown on cheese curd. Cyclopiazonic acid is commonly produced by Penicillium commune and other closely related *Penicillium* species that colonize cheese surfaces, but it is generally not produced or produced only in small quantities by P. camemberti strains used in cheese production (7, 12). Pigment production was also qualitatively described by photographing colonies of each strain grown on CCA. Spore, mycotoxin, and pigment production are traits that are often coregulated by global regulators in *Aspergillus* and *Penicillium* species (19, 20). Mycotoxin production and pigment production are thought to be traits that are important for filamentous fungi to compete with other microbes or tolerate oxidative stress (21, 22); however, these traits are frequently lost within fungal populations, suggesting that they are costly (23). We predicted that adaptation to cheese might lead to the loss of some of these costly traits in the high-resource and reduced-competition environments of cheese rinds.

We observed a general pattern of reduced reproductive output, mycotoxin production, and pigmentation in evolved strains compared to the ancestral strain. Reproductive output was significantly lower in all domesticated phenotypes than in the ancestral strain (ANOVA with Dunnett’s test: *F*_8,18_ = 178.6, *P < *0.001), with some strains having approximately 4-log reductions in CFU produced per area of colony (Fig. 2D). Analysis of a subset of strains spanning the domesticated phenotype continuum demonstrates that the reduced reproductive output per unit area was due in part to a reduction in spore production in domesticated strains (∼80% reduction in spore production compared to the ancestral strain; Fig. S5). We also detected a substantial loss of CPA production across all evolved strains of P. commune 162_3FA (ANOVA with Dunnett’s test: *F*_8,18_ = 38.2, *P < *0.001), with three strains (M6, M7, and M8) having no detectable levels of CPA. Pigment production followed the pattern of loss of reproductive output and CPA production, with intermediate light blue phenotypes that had intermediate levels of reproductive output and CPA production (M2, M9, and M10) and completely white phenotypes having the lowest levels of reproductive output and CPA production (M3, M5, M6, M7, and M8). The observed phenotypic changes were not transient; repeated transfer of domesticated strains on CCA did not lead to reversions to ancestral morphologies (Fig. S6). These trait analyses suggest that coregulated loss of reproductive output, mycotoxin production, and pigmentation had occurred during adaptation of P. commune to the cheese environment.

10.1128/mBio.02445-19.5 FIG S5 Spore production of ancestor and domesticated strains of Penicillium commune 162_3FA. Spores were harvested from plugs taken from the center of fungal colonies and were quantified using a hemocytometer. Bars with the same letter are not significantly different from one another (ANOVA *F*_3,16_ = 105.6, *P < *0.001 with Tukey’s HSD *post hoc* test). Error bars represent 1 standard deviation. *n* = 5. Download FIG S5, DOCX file, 0.1 MB. Copyright © 2019 Bodinaku et al. 2019 Bodinaku et al. This content is distributed under the terms of the Creative Commons Attribution 4.0 International license.

10.1128/mBio.02445-19.6 FIG S6 Stability of Penicillium commune 162_3FA domesticated phenotypes. Domesticated strains were transferred weekly to new cheese curd agar, and colony morphology was photographed. The white morphology was stable over time. Download FIG S6, DOCX file, 0.2 MB. Copyright © 2019 Bodinaku et al. 2019 Bodinaku et al. This content is distributed under the terms of the Creative Commons Attribution 4.0 International license.

The fitness of domesticated strains may differ from that of the ancestral strain growing on the rich cheese medium because domestication may shift resource allocation from costly traits (e.g., secondary metabolite production) to growth. To test whether domesticated strains had a higher level of fitness than the ancestral strain, we used competition experiments where equal amounts of a domesticated strain (M9) were coinoculated with the ancestor. We were able to conduct these competition experiments only with domesticated strains that still had some level of spore production because it is difficult to standardize inputs of ancestor spore producers and white strains that have almost no spore production. As predicted, the domesticated strain outcompeted the ancestral strain after 10 days (Fig. 2E), suggesting that the coordinated loss of traits during domestication leads to higher fitness in the cheese environment.

### Domestication shifts the *Penicillium* volatilome from musty to cheesy aromas.

While working with the domesticated strains, we noticed that their aromas were strikingly different from that of the ancestral strain. The ancestral strain smelled musty and earthy, whereas the domesticated strains smelled fatty and surprisingly reminiscent of aged cheese. The aromas of cheese are volatile organic compounds (VOCs) that are produced by filamentous fungi and other microbes during proteolysis, lipolysis, and other processes that decompose the cheese substrate (11, 24, 25). These VOCs are important determinants of how consumers perceive the quality of a cheese and can create variation in aroma profiles across surface-ripened cheese varieties (26, 27). To quantitatively assess whether domestication of *Penicillium* on cheese alters volatile aroma production, we captured volatiles produced by ancestral and evolved strains of *Penicillium* using headspace sorptive extraction (HSSE) followed by analysis with gas chromatography-mass spectrometry (GC-MS) (28, 29). We compared the ancestral P. commune 162_3FA strain with three domesticated strains—M2, M5, and M6—that spanned the continuum of reproductive output, mycotoxin, and pigment traits (Fig. 2D).

**FIG 3:**
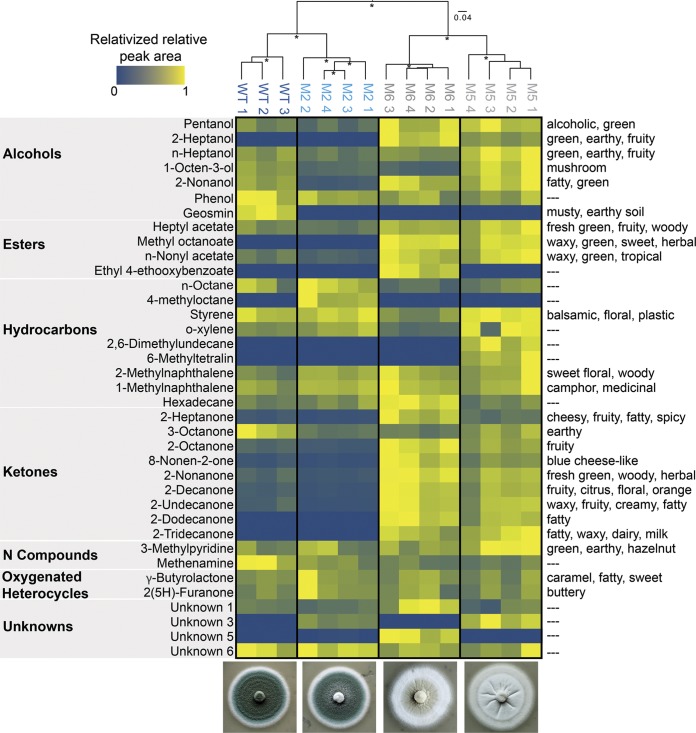
Volatile organic compound (VOC) production of ancestral and domesticated *Penicillium*. Because total concentrations of VOCs are highly variable across different compounds, visualization was simplified by relativizing the relative peak areas from GC-MS chromatograms within each VOC to the highest concentration detected for that VOC. Only the VOCs that were detected across all replicates are shown. See Table S2 for all VOCs and their relative peak area values. The UPGMA tree is clustering the VOC profiles for each replicate based on Bray-Curtis dissimilarity. Asterisks indicate clusters with >70% bootstrap support. WT, ancestor phenotype. M2, M5, and M6 are all domesticated phenotypes. Numbers 1 to 4 after strain designations indicate biological replicates. Because of accidental sample loss during processing, only three biological replicates were collected from the WT. Descriptors on the right represent known aroma qualities of detected VOCs.

As suggested by our preliminary olfactory observations, the composition of volatiles produced by domesticated strains shifted substantially from the ancestor (Fig. 3, ANOSIM *R *= 1.0, *P < *0.001). Geosmin was the only VOC that was produced by the ancestor and was absent in all three of the domesticated strains (6.722% contribution in similarity percentage [SIMPER] analysis of the ancestral strain versus all domesticated strains pooled) (Fig. 3; see also Table S2). Geosmin is widely recognized for contributing a musty aroma to environmental and food samples and is produced by both bacteria and fungi, including *Penicillium* (30–32). It has a very low odor threshold, meaning that even small amounts of geosmin produced by *Penicillium* can be perceived as strong aromas (33). The loss of geosmin production could be the major driver of the perceived loss of musty aromas in domesticated *Penicillium* strains.

10.1128/mBio.02445-19.8 TABLE S2 Overview of all volatile organic compounds detected in the ancestor (ANC) strain and domesticated strains M2, M5, and M6 of Penicillium commune strain 162_3FA. Download Table S2, XLSX file, 0.1 MB. Copyright © 2019 Bodinaku et al. 2019 Bodinaku et al. This content is distributed under the terms of the Creative Commons Attribution 4.0 International license.

In addition to a loss of musty aromas, evolved strains produced larger amounts of methyl ketones and other VOCs associated with molds used in cheese production. Typical Camembert flavor has been defined in patents as containing 2-heptanone, 2-heptanol, 8-nonen-2-one, 1-octen-3-ol, 2-noanol, phenol, butanoic acid, and methyl cinnamate (11). All of these VOCs, except butanoic acid and methyl cinnamate, were detected in our GC-MS profiling, and several were found to be major drivers of differences in VOC profiles of domesticated strains compared to the ancestor (Fig. 3) (2-heptanone = 12.1% SIMPER analysis contribution; 8-nonen-2-one = 8% contribution; 1-octen-3-ol = 7% contribution). Other methyl ketones that have been detected in P. camemberti (34), including 2-nonanone (13.8% contribution) and 2-undecanone (12.3%), were also detected in higher concentrations in evolved strains than in the ancestral strain and contributed strongly to differences in VOC profiles (Fig. 3). These methyl ketones are perceived as emitting the cheesy, fatty, fruity, and green-plant-like aromas that are typically associated with ripened cheeses (35, 36). Collectively, these VOC data demonstrate a dramatic remodeling of the volatilome of P. commune as a result of rapid domestication on cheese.

### Comparative transcriptomics demonstrates global downregulation of secondary metabolite production in domesticated *Penicillium* strains.

To explore additional shifts in metabolic processes in cheese-adapted *Penicillium* not captured by our targeted metabolomics above, we used RNA-seq to compare the global expression patterns of the ancestral P. commune to those of one domesticated strain (M5). This strain was selected because it showed intermediate levels of reductions of reproductive output, CPA production, and pigment production. We predicted that, in addition to shifts in gene expression related to spore and pigment production, genes associated with other secondary metabolites not measured would also be downregulated.

**FIG 4:**
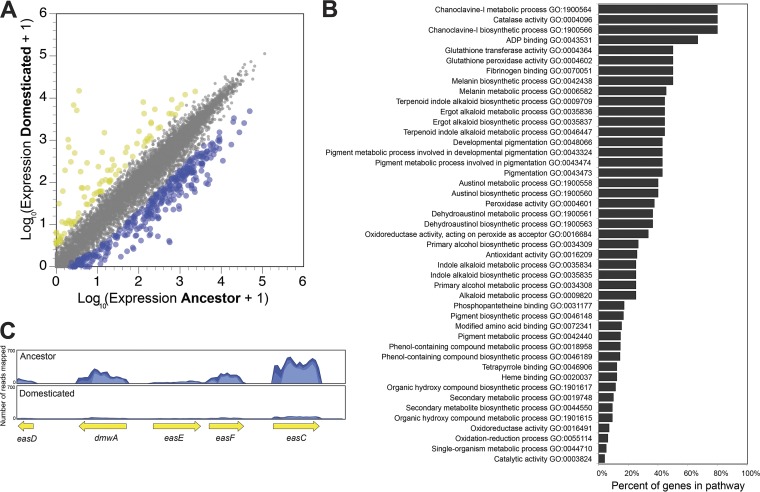
Experimental domestication shifts global gene expression of *Penicillium* on cheese. (A) Differences in gene expression between ancestor and domesticated Penicillium commune 162_3FA. Each dot represents a transcript from across the genome. Yellow dots represent those transcripts that had higher expression, and blue dots represent those transcripts that had lower expression (5-fold change in expression; FDR-corrected *P* value < 0.05). (B) Pathway enrichment analysis showing the distribution of GO terms that were significantly enriched in genes with decreased expression in the domesticated phenotype (strain M5). (C) Representative mapping of reads to the ergot alkaloid synthesis (*eas*) gene cluster.

The transcriptome of strain M5 had 356 genes whose expression levels were significantly lower than those measured for the ancestral strain, or about 3.2% of all protein-coding genes (Fig. 4A). Only 86 genes had higher expression levels in M5 than in the ancestor. An enrichment analysis of gene ontology (GO) terms associated with these differentially expressed genes highlighted the substantial downregulation of secondary metabolite production (Fig. 4B). Many pathways that were significantly enriched in the list of downregulated genes were associated with pigment production (melanin biosynthesis) and production of a range of secondary metabolites, including chanoclavine-I, austinol, and dehydroaustinol (Fig. 4B; see also Table S3). One striking example is the ergot alkaloid synthesis (*eas*) gene cluster. Ergot alkaloids can be toxic to mammals, and recent work demonstrated that genomes of P. camemberti from cheese contain some genes in the ergot alkaloid biosynthesis pathway and can produce some early precursors of ergot alkaloids (37). The *eas* gene cluster is also present in P. commune 162_3FA and is strongly downregulated (a mean of −42-fold change across *dmwA*, *easE*, *easF*, and *easC* genes) in strain M5 compared to the ancestor (Fig. 4C). In addition to the dramatic decrease in the expression of genes associated with secondary metabolite production, the observed reductions in the levels of conidia produced by domesticated strains (Fig. S5) are supported by strong downregulation of *abaA*, which regulates development of condia in *Aspergillus* (38) (Table S3).

10.1128/mBio.02445-19.9 TABLE S3 Overview of genes that were differentially expressed between the ancestor strain and strain M5 of Penicillium commune strain 162_3FA. Download Table S3, XLSX file, 0.3 MB. Copyright © 2019 Bodinaku et al. 2019 Bodinaku et al. This content is distributed under the terms of the Creative Commons Attribution 4.0 International license.

### Genomic evolution does not explain phenotypic and metabolomic traits in domesticated *Penicillium* strains.

To determine if the evolved phenotypes can be explained by genomic changes, including single-nucleotide polymorphisms (SNPs) or insertions or deletions (indels), we sequenced the genomes of five strains (M2, M5, M6, M7, and M9) that span the spectrum of domesticated phenotypes. While several high-confidence SNPs were identified in each strain (Table S4), these SNPs were not associated with genes of known function that could explain the domesticated phenotypes. Strain M2 had a nonsynonymous mutation (G-to-S amino acid change) in a gene with a predicted LeuD-like beta/beta/alpha structure, and strains M6 and M7 had a nonsynonymous mutation (E-to-D) in a gene of unknown function. All strains had a variety of mutations in regions outside predicted genes, but we could not link these mutations to promoters or other regions that might affect gene activity (Table S4). We also searched for mutations in well-characterized global regulators of fungal secondary metabolism and development known to regulate the phenotypic changes that we observed in our evolved strains (*wetA*, *laeA*, *veA*, and *velB*) (39–42), but we did not find mutations in these regions. While these data do suggest that genomic mutations occurred during the evolution experiment, they do not identify a clear genomic explanation for the phenotypic evolution of P. commune in our experimental system.

10.1128/mBio.02445-19.10 TABLE S4 Single-nucleotide polymorphisms detected in the genomes of domesticated strains of Penicillium commune strain 162_3FA. Download Table S4, XLSX file, 0.01 MB. Copyright © 2019 Bodinaku et al. 2019 Bodinaku et al. This content is distributed under the terms of the Creative Commons Attribution 4.0 International license.

### Domesticated phenotypes of *Penicillium* are found at low frequencies in cheese caves.

Our work described above provided experimental evidence that *Penicillium* molds can rapidly domesticate in the cheese environment. But does this domestication process occur under the more realistic conditions of a cheese cave? To answer this question, we deeply sampled a *Penicillium* sp. strain 12 population from a cheese cave in Vermont, USA. The sampling of *Penicillium* sp. strain 12 occurred 4 years after the initial isolation of this strain. We removed patches of the fungus from the surface of 43 different wheels of cheese and plated out each of the patch samples to determine the frequencies of wild-type versus domesticated phenotype colonies. Much of our experimental work was focused on P. commune 162_3FA, and, ideally, we would have sampled a population of this fungus. However, we were unable to find a large enough population of P. commune 162_3FA in the cave where it was originally isolated.

**FIG 5:**
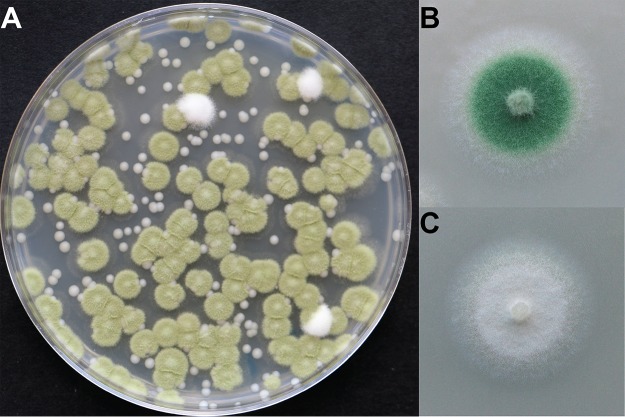
Domesticated phenotypes of *Penicillium* are present in cheese caves. (A) A plate showing *Penicillium* sp. strain 12 isolated from a cheese cave in Vermont, USA. White colonies exist at low frequencies in this fungal population. The small smooth beige colonies represent the yeast Debaryomyces hansenii. (B) Wild-type phenotype of the mold isolated from the population and grown on cheese curd agar. The intensities of the green pigmentation differ between panel A and panel B because panel A shows a fungal population grown on plate count agar and panel B shows a fungal population grown on cheese. (C) A white domesticated strain isolated from the same population as the wild type.

White domesticated phenotype colonies were detected on 12 of the 43 wheels of cheese and were infrequent relative to the wild-type colonies (0.36% frequency) (Fig. 5). This low abundance of domesticated strains in these multispecies rind communities aligns with the low frequency of domesticated phenotypes observed when *Penicillium* sp. strain 12 evolved in the presence of competitors (2.23%) (Fig. S3B). This survey demonstrates that strains with domesticated phenotypes can be detected in caves where cheeses are aged. These rare domesticated strains were likely the source of the original white molds used in industrial Camembert production.

### Conclusions.

Novel ecological opportunities are thought to promote the diversification of plants and animals during adaptive radiations (43–45), and similar processes may occur when wild microbial populations colonize the high-resource environments of fermented foods. Cheese is a resource-rich substrate that provides microbes from natural populations with novel ecological opportunities. Facilities where natural rind cheeses are aged are relatively stable environments where the levels of stressors encountered the natural world, including resource limitation and UV stress, are reduced. While these facilities are carefully managed to keep out pathogens, wild molds from natural populations commonly colonize the surfaces of certain cheeses where a natural rind is desired (6, 7, 14). Using experimental evolution, we demonstrate that cheese aging environments have the potential to promote rapid trait evolution of *Penicillium* species. Changes in the cheese environment that suppressed population size, including competition and decreased resource availability, inhibited trait evolution during domestication. Previous comparative genomic studies of *Penicillium* molds from cheese and fermented meat have identified genomic signatures of evolution over longer timescales in fermented food environments (3, 46). Our experimental work demonstrates that in just a few weeks, *Penicillium* molds can adapt to the cheese environment through the loss of energetically costly traits, including spore production, pigment production, and mycotoxin production.

Previous studies in *Aspergillus* and *Penicillium* species have revealed similar rapid trait change under conditions in which fungi were subcultured in rich laboratory media over many generations (47–49). This phenomenon has been called “degeneration” because desired industrial traits or traits of interest for laboratory studies are lost. These cultures experience a transition from a high-competition and low-resource natural environment (plants, soil, etc.) to a low-competition and high-resource environment (rich laboratory media) similar to the transition that occurs when fungi colonize cheese. For example, serial transfer of Aspergillus parasiticus caused rapid loss of secondary metabolite production, decreased sporulation, and changes in pigment production (48) at a timescale (several weeks) similar to that observed in our work on cheese. A similar pattern of trait degeneration with serial transfer was observed previously with A. flavus (49). Together with our work on *Penicillium*, those studies demonstrated rapid and coordinated phenotypic and metabolomic shifts in a range of filamentous fungi as they were stably maintained in high-resource environments.

One of the most striking changes that we observed in our evolved *Penicillium* strains was a shift in VOC production. Even though the domestication process in our experiments was left undirected, white phenotypes stopped producing the musty VOC geosmin that is generally considered undesirable in foods (31) and increased production of ketones and other VOCs that are considered desirable in Camembert and other cheeses (11, 32). The genes and pathways responsible for production of the secondary metabolite geosmin and other VOCs are not well characterized in cheese *Penicillium* species (50), so we are unable to explicitly link the transcriptomic data with the VOC data. Ketone production by cheese fungi occurs as a result of lipases releasing fatty acids from lipids that are then converted into ketones, alcohols, and other VOCs (25, 50, 51). The observed shifts in VOC production could reflect a generalized loss of secondary metabolite production and increased lipid degradation of the cheese substrate.

We did not identify a specific genetic mechanism controlling the observed trait evolution in *Penicillium*, and a genetic mechanism underlying degeneration of *Aspergillus* cultures has also not been identified. While we did observe SNPs in the genomes of the evolved *Penicillium* strains, these SNPs did not clearly relate to known secondary metabolite production or development pathways that would explain the evolved phenotypes. The rapid trait evolution in *Penicillium* may be explainable not by genomic evolution but by transgenerational epigenetic inheritance, which has been proposed to be important in filamentous fungi (52). Global regulators of genes involved with pigment, toxin, and spore production have been identified in *Aspergillus* and other fungi, and some of these regulators, including the methyltransferase LaeA, have been demonstrated to epigenetically regulate transcription (53, 54). Future work characterizing these evolved strains with epigenomic approaches will more clearly identify the specific genetic and molecular changes driving domestication.

A detailed record of how contemporary P. camemberti strains used in cheese production were derived is not available (13), so we cannot know precisely how and when P. commune was domesticated to become P. camemberti. It is possible that the industrial starter cultures used today were isolated as domesticated phenotypes from cheese caves in Europe. Regardless of how these strains were ultimately acquired, our work demonstrates the potential for *Penicillium* molds to rapidly evolve without intentional selection for desired cheese-making traits. Because we observed similar trait shifts in two different *Penicillium* species, it is possible that domesticated phenotypes of many different *Penicillium* species are continuously evolving in cheese caves around the world. More work characterizing the genomic diversity and distributions of wild and domesticated strains of P. commune and P. camemberti is required to better understand this domestication process. For example, comparing the phenotypes, genomes, and transcriptomes of P. commune populations isolated near cheese facilities to those of the populations within those facilities may further illuminate how the built environment selects for specific fungal traits. It would also be useful to determine if the rapid domestication observed in the two *Penicillium* taxa used in this study is a general phenomenon that occurs across other *Penicillium* species during growth on cheese.

Most strains of P. camemberti used in mold-ripened cheese production originate from Europe, providing a limited palette of textures and flavors. Our laboratory domestication results suggest that new strains of *Penicillium* for cheese production could be generated through intentional and controlled domestication processes. Further work characterizing the genetic mechanisms of evolved traits is needed to confirm that domesticated strains are stable across the cheese production process and would yield safe and high-quality cheese.

## MATERIALS AND METHODS

### Isolation and manipulation of *Penicillium* cultures.

Two non-starter *Penicillium* strains, *Penicillium* sp. 162_3FA and *Penicillium* sp. 12, were used in the experiments performed throughout this study. Both fungal strains were isolated from the surface of a natural rind cheese produced and aged in Vermont, USA. Cheeses inoculated with a P. camemberti starter culture had not been placed in the vault where this cheese is aged. A third non-starter mold, *Penicillium* sp. MB, was isolated from a natural rind cheese production and aging facility in California. It was sequenced as part of this work and included in the phylogenomic analysis but was not used in the experimental portion of the paper because its phenotypic scoring results were not as unambiguous as those determined with the other two strains. To determine the putative taxonomic identity of these two molds, whole-genome sequences were obtained using Illumina sequencing as previously described for a *Mucor* isolate (55). Genomes were assembled using the *de novo* assembler in CLC Genomics Workbench and annotated using GenSAS (https://www.gensas.org/).

### Phylogenomic analysis of *Penicillium* strains.

To reconstruct the evolutionary relationships among the described *Penicillium* species and the isolates used in this work, we used a recently published phylogenomic approach (56). We obtained a comprehensive set of genomes from *Penicillium* species using NCBI’s Taxonomy Browser. We downloaded the 33 available *Penicillium* genomes on 5 February 2018. In addition to the 33 *Penicillium* genomes, we also downloaded three genomes from representative species in the genus *Aspergillus* for use as outgroup taxa. Altogether, our data set contained a total of 39 taxa—3 *Penicillium* genomes sequenced in the present study, 33 publicly available *Penicillium* genomes, and 3 *Aspergillus* taxa.

To identify orthologous genes, we used Benchmarking Universal Single-Copy Orthologs (BUSCO; version 2.0.1 [57]) pipeline and the Pezizomycotina database (creation date 13 February 2016) from OrthoDB, version 9 (58). Using 3,156 universal single-copy orthologs from the Pezizomycotina database (or BUSCO genes), we observed that the *Penicillium* sp. strain MB, *Penicillium commune* strain 162_3FA, and *Penicillium* sp. strain 12 isolates had 3,099/3,156 (98.2%), 3,097/3,156 (98.2%), and 3,096/3,156 (98.1%) BUSCO genes present in their genomes, respectively. To construct the phylogenomic data matrix, we first retained only those BUSCO genes that were present as single copies in at least 20 taxa (i.e., >50% taxon occupancy). The 3,111 groups of BUSCO orthologous genes were concatenated into a single phylogenomic data matrix that contained 5,498,894 sites and had a taxon occupancy rate of 97.65% ± 5.23%. The methods used for construction of the phylogenomic data matrix and gene-based maximum likelihood schemes of concatenation and coalescence were previously described in detail (56). Examination of the resulting phylogeny revealed high concordance with previous whole-genome-based analyses with full support at every internode using UFBoot and local posterior probability, with the exception of the split of sections *Exilicaulis* and *Lanata-divaricata*, which received a local posterior probability value of 0.97. Importantly, our analyses place the newly sequenced Penicillium commune strain in the section of *Fasciculata* (see Fig. S1A in the supplemental material).

### Experimental evolution of *Penicillium* on cheese.

Each of the *Penicillium* strains was grown in our experimental cheese system, which consisted of 20 ml of CCA in a standard 100-by-15-mm Petri dish. CCA is composed of freeze-dried unsalted cheese curd from a blue cheese produced in Vermont (100 g/liter), xanthan gum (5 g/liter), salt (30 g/liter), and agar (17 g/liter). CCA allows controlled manipulations of cheese rind communities, and growth on CCA accurately mimics the dynamics of cheese rind development (14). To start the evolution experiment, each strain was initially inoculated with 500 CFU across the surface of the CCA plate. Each experimental cheese community was incubated for 7 days in the dark at 24°C and 95% humidity. Experimental communities were serially transferred to new CCA every week for a period of 8 weeks.

To manipulate the biotic environment throughout the evolution experiment, cheese rind bacteria and yeasts were added to four replicate communities to create a “*Penicillium* + community” treatment. Yeast strain Debaryomyces hansenii 135B and bacterial strains Staphylococcus xylosus BC10 and Brachybacterium alimentarium JB7 were added at the same density as *Penicillium* at the initial inoculation. We selected these three microbial species for the *Penicillium*-plus-community treatment because they represent taxa that are common members of natural rind cheese microbiomes (14, 16, 55), and we stably maintained all three species during the duration of the experiment. We acknowledge that these community members may have evolved during the experimental domestication experiment, and we did not attempt to control for their evolution throughout the experiment.

To manipulate total resource availability throughout the evolution experiment, we created a “low-cheese” treatment environment which consisted of the same components as CCA except 10 g/liter of freeze-dried unsalted cheese curd (instead of 100 g/liter as found in “normal cheese”) was used in the medium. The pH of the “low-cheese” treatment was identical to that of “normal cheese.” In the “alternating normal/low” treatment, we alternated transfers each week between full-strength and dilute CCA, starting with full-strength CCA when setting up the experiment.

At each transfer, the CCA from each community was removed from the Petri dish and homogenized inside a Whirl-Pak bag containing 30 ml of 1× phosphate-buffered saline (PBS). From this homogenized mixture, an aliquot of 100 μl was plated onto new CCA to seed a new community. The aliquot that was transferred to form a new population represented 0.3% of the previous population (see Fig. S2 and S3A for population sizes). Another aliquot was serially diluted and plated onto PCAMS (plate count agar supplemented with 0.1% milk and 1% salt) with 50 mg/liter of chloramphenicol (to inhibit bacterial neighbors in the “*Penicillium* + Community” treatment) for colony counting and scoring colony phenotypes. Glycerol stocks were made at each transfer so that communities could be archived and revived later if needed.

Phenotypic evolution was tracked by scoring wild-type and domesticated phenotype colonies at each transfer. Domesticated phenotype colonies were considered to have differences with respect to pigment intensity, distribution of pigment around the colony, colony texture, degree of sporulation (inferred from dusty versus smooth appearance of colony), and extent of mycelium production (explained in detail in Table S1A to D). Phenotyping was done after 5 days of incubation of PCAMS plates containing the output of each transfer. PCAMS output plates were incubated at 24°C for 5 days before phenotyping was completed. Phenotyping occurred on plates with at least 100 colonies.

### Reproductive and mycotoxin trait analysis.

Reproductive and mycotoxin traits were measured only for the ancestor strain and selected evolved strains of *Penicillium* sp. 162_3FA because it is most closely related to P. camemberti. The following strains were used in these assays: ancestor, M2, M3, M5, M6, M7, M9, and M10. These strains were selected for trait profiling because they spanned the spectrum of visible colony types, ranging from similar to wild type (although slightly less blue) to completely white (Table S1A and B). To determine levels of reproductive output, each strain was inoculated on three replicate plates at a density of 50 CFU on the surface of 20 ml of CCA in a standard 100-by-15-mm Petri dish. At this density, individual CFU were discernible. Plates were incubated for 7 days at 24°C and 95% humidity. From three individual colonies, a sterile circular cork borer with a diameter of 0.7 cm was inserted into the center of the colony. The excised colony plug was serially diluted, and CFU were determined on PCAMS. Reproductive output was expressed as CFU count per square centimeter.

For a subset of strains (ancestor, M5, M6, and M9), we measured the level of production of spores (conidia) per area of fungal mycelium to confirm that the trends in reproductive output were partly driven by changes in spore production. A 20-μl volume of inoculum from a frozen stock was spotted onto replicate 100-by-15-mm Petri dishes containing 20 ml of CCA. After 6 days of growth at 24°C, a 0.6 cm wide cork borer was used to remove a plug of mycelium directly from the center of a colony. This plug was homogenized in 200 μl of 1× PBS, and spore density was determined using a hemocytometer. Data were expressed as the number of spores per square centimeter, and differences in spore production were assessed with ANOVA and Tukey’s honestly significant difference (HSD) *post hoc* test.

To determine how the level of production of the mycotoxin CPA changed in evolved strains compared to ancestors, we measured CPA production in the following strains: ancestor, M2, M3, M5, M6, M7, M9, and M10. A total of 40,000 CFU of each strain was spread across the surface of 20 ml of cheese curd in a 100-by-15-mm Petri dish. Three biological replicates of each strain were used in the experiments. Plates were incubated in the dark for 3 days at 24°C and 11 days at 4°C. After the 14-day incubation, the medium was harvested from the plate, placed into a Whirl-Pak bag, and homogenized. Samples were frozen at –80°C until analysis.

The CPA concentration of the CCA was measured using liquid chromatography with tandem mass spectrometry (LC-MS/MS) at Romer Labs (Union, MO, USA). The homogenized cheese curd sample was extracted in a 50/50 mixture of acetonitrile and deionized water by shaking for 90 min. The supernatant was filtered, and 10 ml was mixed with 500 μl of acetic acid. A 1-ml volume of this solution was subjected to vortex mixing in a MycoSpin column (Romer Labs) for 1 min and was centrifuged for 30 s at 10,000 rpm. A 75-μl volume of the purified extract was injected into a Shimadzu high-performance liquid chromatography (HPLC) system with a Phenomenex Gemini HPLC C_18_ column (4.6 by 150 mm, 5-μm pore size), with mobile phase A consisting of electrospray ionization (ESI) performed with 5 mM ammonium formate–0.1% formic acid–deionized water and mobile phase B consisting of acetonitrile. The injection volume was 40 μl, the flow rate was 1.0 ml/min, and the column temperature was 40°C. Internal standards of CPA were used to construct a calibration curve.

Stability of traits was assessed in two evolved strains, M5 and M6. Three replicate plugs of each of these strains were transferred to fresh CCA weekly using heat-sterilized stainless steel cork borers (6-mm diameter). Colonies were photographed at each transfer as described for Fig. S6.

### Competition experiments.

We competed ancestor *Penicillium* sp. 162_3FA with the evolved strain M9 to determine whether strains with domesticated phenotypes have higher fitness than the ancestor strain. It was challenging to standardize input densities of the white strains because they produced fewer spores than the ancestor strain. Strain M9 of *Penicillium* sp. 162_3FA was chosen as a competitor because it still produced significant numbers of spores, making it possible to produce comparable initial inocula of the ancestor and evolved strains. Experiments were conducted in 96-well plates with 150 μl of 10% CCA added to each well and 200 CFU of each strain added at the start of the experiment. Six replicate experimental cheese communities containing the ancestor and evolved strain mix were incubated in the dark at 24°C for 10 days. To determine the abundances of the ancestor and M9 strains at the end of the experiment, each replicate community was removed from the 96-well plate, homogenized in 600 μl 1× PBS, and serially diluted onto PCAMS, and then the ancestor and M9 colonies were counted.

### Volatile profiling.

Cheese volatiles were collected from fungal cultures by headspace sorptive extraction (HSSE) using a glass-encapsulated magnetic stir bar coated with a 0.5-mm-thick layer of polydimethylsiloxane (PDMS). Before each sample was collected, the stir bars were heated from 40°C to 300°C at 5°C/min and flushed with 50 ml/min nitrogen (Airgas) to desorb sorbed organics using a TC2 tube conditioner (Gerstel, Baltimore, MD). HSSE is an equilibrium-driven, enrichment technique in which 10-mm-long stir bars (Twister [Gerstel]) were suspended 1 cm above the sample by placing a magnet on the top side of the collection vessel cover. All cultures were sampled in quadruplicate (*n* = 4) for 4 h. One replicate of the ancestor was lost during sample processing. After collection, the stir bar was removed and spiked with 10 ppm ethylbenzene-d_10_, an internal standard obtained from Restek Corporation (Bellefonte, PA). Organics were introduced into the gas chromatograph/mass spectrometer (GC/MS) by thermal desorption. In addition to Twister blanks, analyses of the agar media were performed to ensure that levels of compounds representing background interference were minimal. If present, the data corresponding to these compounds were subtracted from the fungal data.

Analyses were performed using a model 7890A/5975C GC/MS (Agilent, Santa Clara, CA) equipped with a 30-m-by-250-μm HP5-MS column (0.25-μm pore size). The instrument was equipped with an automated multipurpose sampler (MPS), a thermal desorption unit (TDU), and a CIS4 programmable-temperature vaporizer (PTV) inlet from Gerstel. The TDU, operating in splitless mode, transferred the sample from the stir bar to the CIS4, which was held at –100°C, by ramping the temperature from 40°C to 275°C at 720°C/min, and was then held at an isothermal level for 3 min under conditions of 50 ml/min helium gas flow. Once transferred, the CIS4 was heated from –100°C to 280°C at 12°C/min and was then held at that temperature for 5 min. The GC temperature was held at 40°C for 1 min and then ramped to 280°C at 5°C/min and held for 5 min. The MS was used to scan from 40 to 250 *m/z*, with the electron ionization (EI) source maintained at 70 eV. A standard mixture of C7 to C30 n-alkanes, purchased from Sigma-Aldrich (St. Louis, MO), was used to calculate the retention index (RI) of each compound in the sample.

Ion Analytics (Gerstel) spectral deconvolution software was used to analyze the GC/MS data. Peak identification was performed through comparison of sample and reference compound spectral patterns and retention indices using NIST05, Adams Essential Oil Library, and literature. Compound identification was performed on the basis of the following set of conditions. First, peak scans were required to be constant for five or more consecutive scans (differences of ≤20%). Second, the level of scan-to-scan variance (SSV, or relative error) was required to be <5. The SSV represents relative error levels calculated by comparing the mass spectrum at a given peak scan to the mass spectrum at another. The smaller the difference, i.e., the closer the SSV is to zero, the better the MS agreement. Third, the Q-value was required to be ≥93. The Q-value is an integer between 1 and 100; it measures the total ratio deviation of the absolute value calculated by dividing the difference between the expected and observed ion ratios by the expected ion ratio and multiplying the result by 100 for each ion across the peak. The closer the value is to 100, the higher the certainty of the accuracy of the result of the comparison between database and sample spectra. Finally, the Q-ratio represents the ratio of the molecular ion intensity to confirmatory ion intensities across the peak; it also must be ≤20%. When all criteria are met, the software assigns a compound name or numerical identifier to the peak from the database.

To cluster the VOC data, an unweighted pair group method using average linkages (UPGMA) tree with 100 bootstraps was constructed using a Bray-Curtis dissimilarity matrix in PAST3. Analysis of similarity (ANOSIM) was used to test whether there were differences between the ancestral strain and the evolved strain in VOC profiles. ANOSIM *R* values indicate the degree to which groups are separate, with a value of 1 representing complete separation and a value of 0 indicating a complete lack of separation. Similarity percentage (SIMPER) analysis of Bray-Curtis dissimilarity distances was used to identify the compounds that contributed most to differences in VOC profiles.

### RNA sequencing.

To determine global changes in gene expression in cheese-adapted *Penicillium*, we compared the transcriptomes of the ancestor and one evolved strain (M5) of *P. commune* 162_3FA. Inocula of both strains came from 1-week-old streaks of the fungi growing on PCAMS medium. A 1-cm^2^ plug was taken from the leading edge of mycelium and then homogenized in 500 μl of 1 × PBS. At three evenly spaced locations on a 100-cm-wide Petri dish containing 20 ml of CCA 20 μl of the inoculum was spotted onto the agar surface. After 72 h of growth in the dark at 24°C, the spots were 1.5 cm in width. The ancestor had produced spores and was blue in color, and evolved strain M5 was white in color. The entire fungal mass from each of the three spots was cut away from the CCA and then placed in RNAlater (Qiagen) and stored at –80°C. Four biological replicates were sampled for each of the two strains.

RNA was extracted from one of the three spots from each replicate plate using a Qiagen RNeasy plant minikit after the sample was ground in liquid nitrogen with an autoclaved mortar and pestle. Approximately 100 mg of ground fungal biomass was placed in 750 μl of RLT buffer (Qiagen) with 10 μl of β-mercaptoethanol per 1 ml added to the RLT buffer. The manufacturer’s recommended protocol was followed for RNA extraction, including an on-column DNase treatment. To isolate mRNA, a NEBNext poly(A) mRNA magnetic isolation module (New England Biolabs) was used. This mRNA was used to generate RNA-seq libraries using a NEBNext Ultra II RNA library prep kit for Illumina following the manufacturer’s recommended protocol. The RNA-seq libraries were sequenced using 125-base-pair length, paired-end Illumina sequencing on a HiSeq system at the Harvard Bauer Core.

After trimming of low-quality sequences and removal of failed reads using CLC Genomics Workbench, sequencing yielded 3.5 to 22 million forward reads that were used for read mapping and differential expression analysis. Reads were mapped to a reference genome of P. commune 162_3FA that was sequenced using paired-end 125-base-pair-length Illumina sequencing, assembled with CLC Genomic Workbench *de novo* assembler, and annotated using GenSAS (https://www.gensas.org/). Read mapping was performed with the CLC Genomics Workbench RNA-seq analysis pipeline with the following settings: mismatch cost of 2, insertion cost of 3, deletion cost of 3, length fraction of 0.8, and similarity fraction of 0.8. The number of unique reads mapped (mapped to one specific gene and not additional locations in the genome) was used to determine expression levels, and quantile normalization was used to take into account different levels of sequencing across replicates. Other methods of calculating gene expression and normalization (e.g., calculation of reads per kilobase per million [RPKM]), were assessed and the results did not change the main findings of the differential expression analysis. Identification of genes that were differentially expressed in the evolved strain compared to the ancestor was completed by using the empirical analysis of differential gene expression tool in CLC Genomics Workbench. This pipeline uses the exact test for two-group comparisons. We considered those genes with greater than 5-fold change in expression and false-discovery-rate (FDR)-corrected *P* values of <0.05 to represent differentially expressed genes. To identify specific biological pathways that were enriched in the sets of downregulated or upregulated genes, we used KOBAS 2.0 to conduct a hypergeometric test on functional assignments from the gene ontology (GO) database (using the Aspergillus flavus genome as a reference for GO identifier [ID] assignment) with Benjamini and Hochberg FDR correction.

### Resequencing genomes of *Penicillium* sp. 162_3FA domesticated strains.

To identify SNPs or indels that might explain domesticated phenotypes, we resequenced genomes of several domesticated strains, namely, strains M2, M5, M6, M7, and M9. DNA of each strain was extracted from 7-day-old cultures grown on PCAMS media. Each strain was resequenced to ∼15× to 20× coverage using paired-end 100-base-pair read libraries sequenced on an Illumina HiSeq 2000 system as described above. Reads were mapped to the draft *Penicillium commune* 162_3FA genome assembly using end-to-end read alignment in Bowtie 2. Variants were detected in both coding and noncoding regions using two different variant calling programs: FreeBayes v 1.1.0 and the variant finder in Geneious 11.0.5. Using both approaches, variants were called only when there was 10× coverage at a region of the genome and when the variant frequency was 100%. Read stacks of SNPs or indels that were called using both programs were manually inspected to confirm variant calls.

### Cheese cave population population sampling.

Sterile toothpicks were used to sample rinds of 43 wheels of a natural rind blue cheese in the same caves where *Penicillium* sp. strain 12 had been previously isolated. Samples were placed in 1× PBS and stored at 4°C for 24 h, and then individual samples from a wheel of cheese was plated onto PCAMS with chloramphenicol to inhibit bacterial growth. Plates were incubated at 24°C for 7 days before assessing plates for the presence of white domesticated phenotypes. Camembert-style cheeses inoculated with P. camemberti are aged in physically separated vaults at the same facility. To confirm that the strains with white domesticated phenotypes were derivatives of wild-type *Penicillium* sp. strain 12 and did not represent contamination from starter cultures, we used whole-genome sequencing as described above to sequence a white domesticated phenotype isolate. Read mapping using Bowtie 2 revealed 99.9% pairwise identity of the genome with the reference genome of *Penicillium* sp. strain 12.

### Data availability.

Whole-genome sequences of Penicillium commune strain 162_3FA and *Penicillium* sp. strain 12 have been submitted to NCBI under accession no. MUGJ00000000 and MUGI00000000, respectively. Raw data from RNA sequencing of the *Penicillium* sp. 162_3FA strain ancestral strain and *Penicillium* sp. 162_3FA strain M5, resequencing of the *Penicillium* sp. 162_3FA evolved strains, and resequencing of the cave isolate of *Penicillium* 12 sp. have been deposited in the NCBI Sequence Read Archive (SRA) under accession no. PRJNA510622.
