# Supplementary material for: Rapid Phenotypic and Metabolomic Domestication of Wild Penicillium Molds on Cheese
Source: mBio. 2019 Oct 15;10(5):e02445-19. doi: 10.1128/mBio.02445-19 (PMC6794487; doi:10.1128/mBio.02445-19)

**Figure S2: Population size of *Penicillium commune* 162_3FA when evolved alone and with a community of cheese microbes.** Lines connect points representing mean colony forming units (CFUs) of four replicate populations and error bars represent one standard deviation of the mean. Total CFUs in the *Penicillium* + community treatment was significantly different from *Penicillium* alone (repeated-measures ANOVA *F*_1,6_= 10.3, *p* = 0.02).
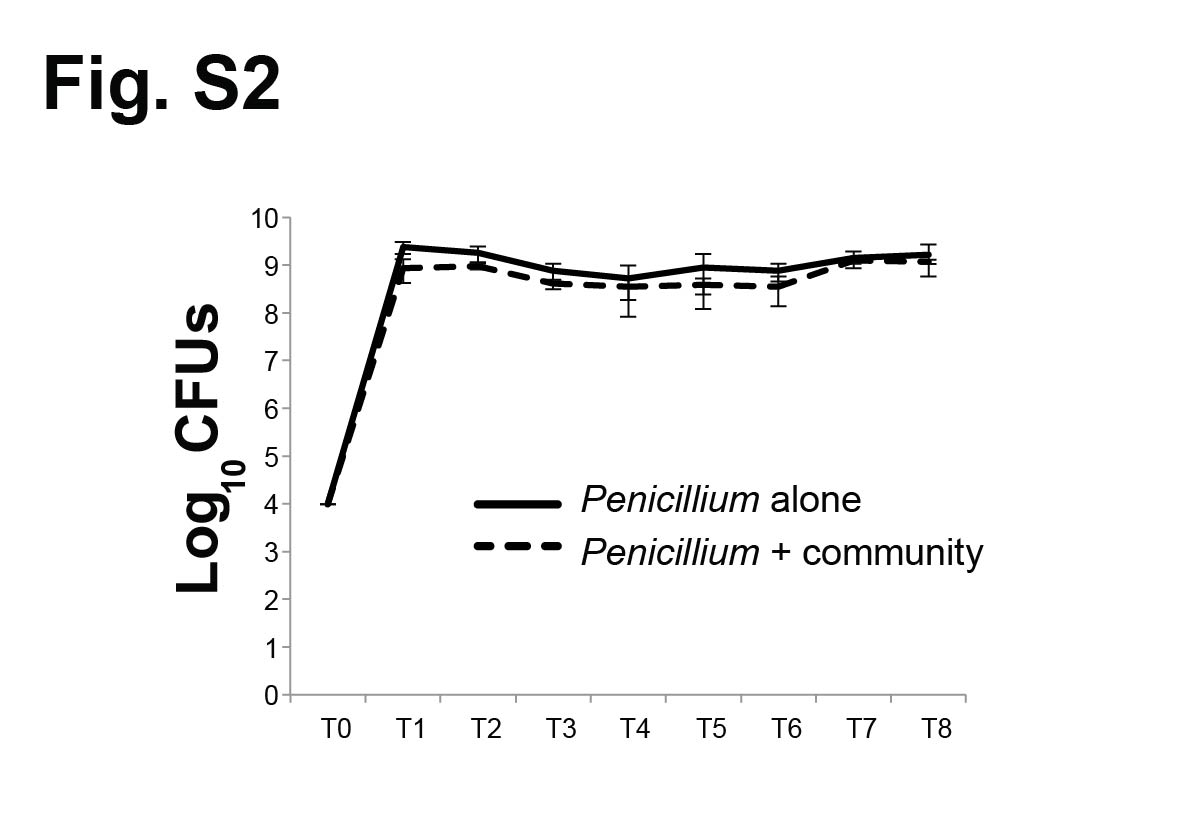

Supplement: FIG S2 [file mBio.02445-19-sf002.docx]
