# Supplementary material for: Rapid Phenotypic and Metabolomic Domestication of Wild Penicillium Molds on Cheese
Source: mBio. 2019 Oct 15;10(5):e02445-19. doi: 10.1128/mBio.02445-19 (PMC6794487; doi:10.1128/mBio.02445-19)
Supplement: FIG S4 [file mBio.02445-19-sf004.docx]

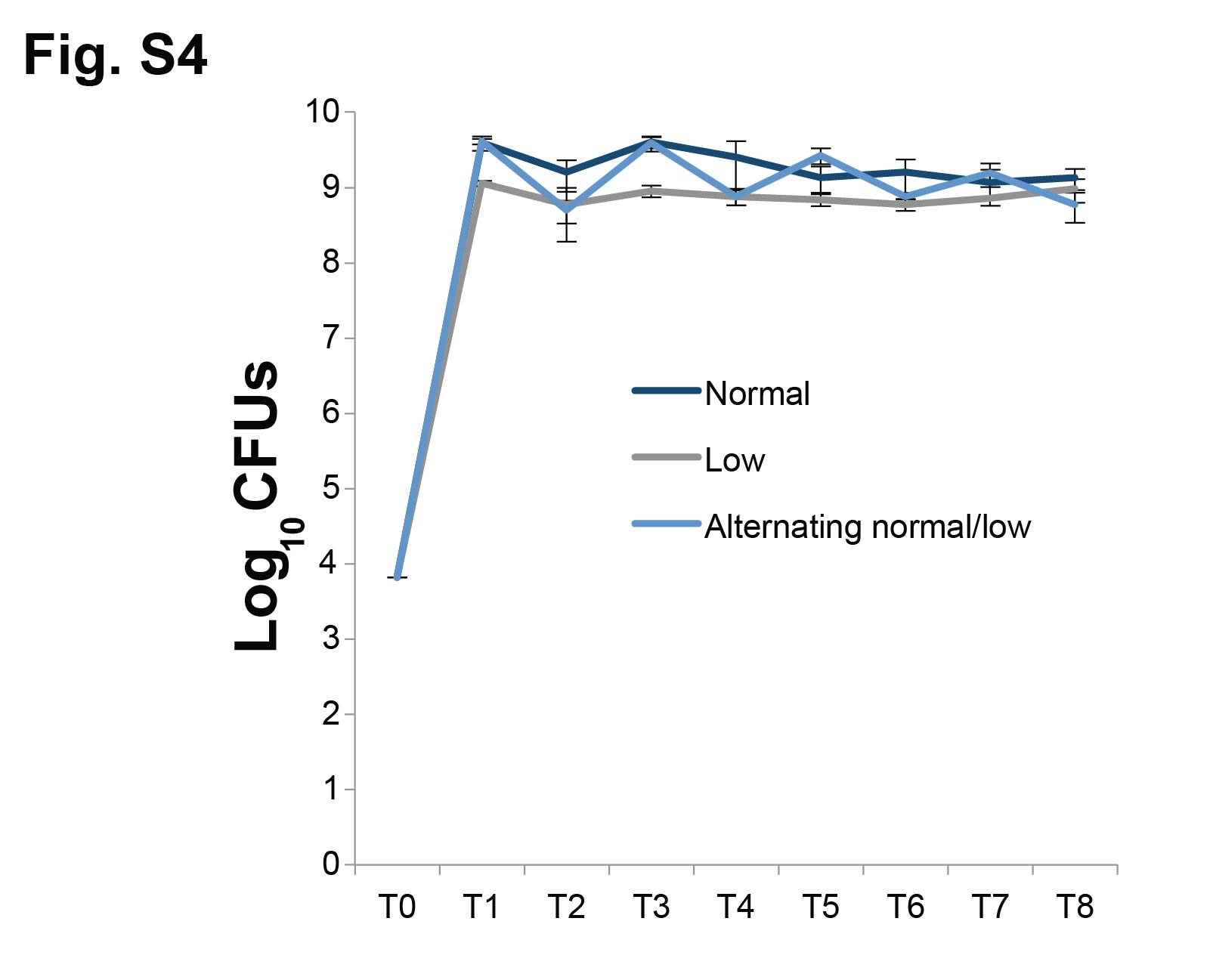


**Figure S4: Population size of *Penicillium commune* 162_3FA when evolved in different cheese nutrient environments.** “Normal cheese” = 10% cheese curd in agar medium. “Low cheese” = 1% cheese curd in agar medium. “Alternating normal/low” = alternating 10% and 1% cheese curd at each transfer. The “Low cheese” treatment suppressed population size (repeated-measures ANOVA *F*_2,9_= 105.1, *p* <0.0001, with Tukey’s HSD post-hoc tests). Lines connect points representing mean colony forming units (CFUs) of four replicate populations and error bars represent one standard deviation of the mean.
