# Supplementary material for: Rapid Phenotypic and Metabolomic Domestication of Wild Penicillium Molds on Cheese
Source: mBio. 2019 Oct 15;10(5):e02445-19. doi: 10.1128/mBio.02445-19 (PMC6794487; doi:10.1128/mBio.02445-19)
Supplement: FIG S5 [file mBio.02445-19-sf005.docx]

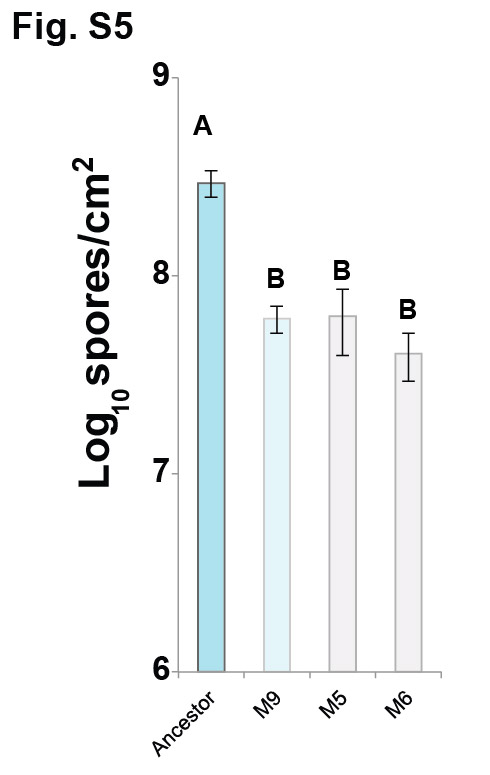


**Figure S5: Spore production of ancestor and domesticated strains of *Penicillium commune* 162_3FA.** Spores were harvested from plugs taken from the center of fungal colonies and were quantified using a haemocytometer. Bars with the same letter are not significantly different from one another (ANOVA *F*_3,16_=105.6, *p*<0.001 with Tukey’s HSD post-hoc test). Error bars represent one standard deviation. n = 5.
