# Supplementary material for: Rapid Phenotypic and Metabolomic Domestication of Wild Penicillium Molds on Cheese
Source: mBio. 2019 Oct 15;10(5):e02445-19. doi: 10.1128/mBio.02445-19 (PMC6794487; doi:10.1128/mBio.02445-19)
Supplement: FIG S6 [file mBio.02445-19-sf006.docx]

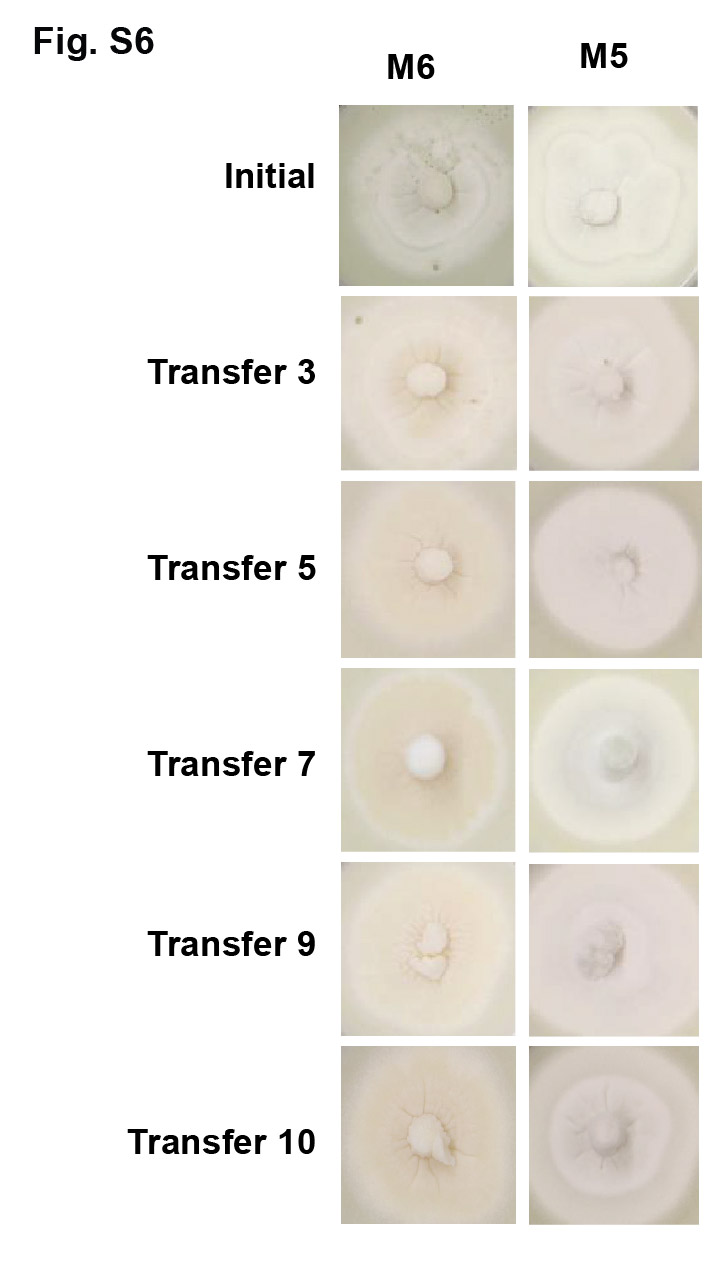


**Figure S6: Stability of *Penicillium commune* 162_3FA mutant phenotypes.** Mutants were transferred weekly to new cheese curd agar and colony morphology was photographed. The white mutant morphology was stable over time.
